# Peer Review of “Impact of Modifiable Risk Factors on the Occurrence of Cutaneous Leishmaniasis in Diyala, Iraq: Case-Control Study”

**DOI:** 10.2196/31514

**Published:** 2021-07-30

**Authors:** Rihana Taher

**Affiliations:** 1 Yemen Field Epidemiology Training Program Ministry of Public Health and Population Sana'a Yemen

**Keywords:** cutaneous leishmaniasis, outbreak, Iraq, risk factors, risk, disease, infectious disease, disease prevention, prevention

*This is a peer-review report submitted for the paper “Impact of Modifiable Risk Factors on the Occurrence of Cutaneous Leishmaniasis in Diyala, Iraq: Case-Control Study.”*

## Round 1 Review

### General Comments

This paper [1] is about a leishmania outbreak, which is a very important public health problem in developing countries.

### Specific Comments

#### Major Comments

1. References need to be rewritten as per the IJMR guide.

2. There are grammar issues.

#### Minor Comments

3. In the Abstract and the *Discussion* section, I suggest you merge the recommendations with the conclusion, and the same in the Discussion…try to make the recommendations bullets in the Discussion.

4. Please don’t use “we” in the beginning of the *Methodology* section.

## Round 2 Review

### General Comments

This paper about a leishmania outbreak in Iraq [1] is talking about a very important health problem in this region and other developing countries.

### Specific Comments

#### Minor Comments

1. Please change the title of the *Background* section to “Introduction.”

2. All tables are not organized, so please delete empty rows and use bold style for table titles.

3. I suggest that you group the references instead of writing each reference separately, eg, (1-4), not (1) then (2) then....

4. Proofreading is highly recommended.
